# Integrating mental health into primary care: evaluation of the Health Action for Psychiatric Problems In Nigeria including Epilepsy and SubstanceS (HAPPINESS) pilot project

**DOI:** 10.1186/s12913-022-07703-1

**Published:** 2022-03-12

**Authors:** Casey Chu, Nichole Roxas, Chinyere M. Aguocha, Emeka Nwefoh, Katie Wang, Charles Dike, Theddeus Iheanacho

**Affiliations:** 1 Yale School of Public Health, New Haven, CT USA; 2 Department of Psychiatry, Yale School of Medicine, New Haven, CT USA; 3 Department of Medicine, Imo State University, Owerri, Imo State Nigeria; 4 CBM International, Country Office, Abuja, Nigeria

**Keywords:** Nigeria, mhGAP, Pilot, Implementation, Stigma, Feasibility, Training, Primary care

## Abstract

**Background:**

The Health Action for Psychiatric Problems In Nigeria including Epilepsy and SubstanceS (HAPPINESS) project trains non-specialist and primary health care workers in Imo State, Nigeria. This project adapted the World Health Organization’s Mental Health Gap Action Programme-Intervention Guide (mhGAP-IG), emphasizing stigma reduction among trainees. This convergent mixed-methods proof-of-concept study evaluates the HAPPINESS pilot project mhGAP-IG training’s impact on mental illness stigma among trainees and barriers, facilitators, and opportunities to consider for project improvement.

**Methods:**

Trainees (*n* = 13) completed a 43-item questionnaire before and after their 5-day training to assess perceptions of mental disorders and attitudes towards people with mental illness. These responses were analyzed using paired-sample t-tests for four subscales of the questionnaire: acceptance of *socializing* with people with mental illness, *normalizing* activities and relationships with people with mental illness, *supernatural causation* of mental illness, and endorsement of a *biopsychosocial* approach to mental illness. Semi-structured key informant interviews (*n* = 11) with trainees, trainers, and local health officials who participated in or supported the HAPPINESS project were thematically analyzed to understand their experiences and perspectives of the project’s barriers, facilitators, and opportunities.

**Results:**

Trainees showed significant improvements on *socializing, normalizing, and supernatural causation* subscales of the stigma questionnaire (*p* < 0.05). No significant effect was seen on the *biopsychosocial* subscale; however, evidence of biopsychosocial beliefs was found in interview responses. Key informant interviews revealed that the HAPPINESS project enhanced trainees’ diagnostic and treatment abilities, mental health awareness, and empathy towards patients. Misinformation, stigma, inadequate funding, and lack of road access to clinics were identified as barriers to mental health care integration into general care in Imo State. Respondents also suggested ways that the HAPPINESS project could be improved and expanded in the future.

**Conclusions:**

This study adds to the limited evidence on the implementation of mhGAP-IG in Nigeria. Using mixed methods, it evaluates how mhGAP-IG can impact perceptions and knowledge of stigma among primary care trainees. It also highlights barriers, facilitators, and opportunities to consider for project growth. Future efforts should focus on clinical support, supervision, health outcomes, as well as scaling up and assessing the cost-effectiveness of the HAPPINESS project intervention.

**Supplementary Information:**

The online version contains supplementary material available at 10.1186/s12913-022-07703-1.

## Introduction

The prevalence of mental, neurological, and substance use (MNS) disorders is on the rise globally [1]. These rates are comparable between high-income countries and low-and-middle-income countries (LMIC’s). However, LMIC’s often have healthcare systems with far less capacity to address such conditions [2]. Nigeria, Africa’s most populous nation and an LMIC [3], has over 7 million people with depression and over 4 million people with anxiety disorders (the highest number of cases compared to other countries in the African region) [4]. The published national lifetime prevalence of any mental disorder in Nigeria is approximately 12% (although experts have estimated this to be 20–30% in actuality) [5], with some regional studies indicating even higher numbers [6–8]. The prevalence of neurological disorders vary by condition, but epilepsy, for instance, has been estimated at 8 per 1000 people [9]. Substance-use disorders have been estimated between 20 and 40% among youths and 14% in the general population [10].

Despite these dire numbers, only about 10% of Nigerians with mental disorders receive any formal specialist mental health care irrespective of severity [5]. Poor and underserved populations are particularly vulnerable to the adverse effects of mental disorders, experiencing a more severe impact on functioning, having drastically limited access to early intervention, and discontinuing treatment due to cost [11]. Significant factors limiting access to formal mental health care in Nigeria include the scarcity of mental health specialists, with only 0.15 psychiatrists per 100 000 of the population (compared to the US’s 10.54 per 100 000) [12], a lack of community-level care for mental disorders [13], the limited integration of mental health integration in primary care [14], and the pervasive stigma towards mental disorders, even among health care providers [15, 16]. In Nigeria, stigma has been widely documented as a deterrent to help-seeking [17–20]. Self-stigma (internalized mental illness stigma leading to diminished self-esteem and self-efficacy) and public stigma (the prejudice and discrimination endorsed by the general population that affects a person) about mental illness are pervasive [21, 22]. Studies using validated and widely used measures of stigma, attitudes, and beliefs across different parts and populations in Nigeria show that the most common understanding of the causation of mental illness was supernatural causes (magic, witchcraft, sorcery, and divine punishment). Social distance and isolation are the most common attitudes, and traditional (unorthodox) medicine is a major treatment preference [23]. There are also stigmatizing beliefs and myths that perpetuate the idea that mental disorders are untreatable [24]. Stigmatizing and negative attitudes toward mental disorders and people with mental illness are also common among health care professionals in Nigeria, which can adversely impact the therapeutic alliance and clinical outcomes [16, 25, 26]. However, to date, only three Nigerian States (Lagos, Osun, and Ogun) have implemented state-wide mental health training for primary health care workers, which are the first contact for people with signs of mental illnesses [27–30].

In accordance with emerging evidence and WHO recommendations, Nigeria’s Mental Health Policy (adopted in 1991 and reviewed since) established that mental health care was to be integrated into primary healthcare, with responsibility for implementation delegated to local governments [31]. This policy, however, was poorly implemented due to insufficient training and supervision of primary care workers, funding, and political will [32]. A revision of this policy in 2013 gave rise to the Policy on Mental Health Service Delivery. It recognized the nuances of integrating mental health care within the primary healthcare system and outlined specific recommendations for primary, secondary, and tertiary care. For instance, for primary care, the policy recommended that primary care centers (PHCs) have a reliable stock of psychiatric medications and strengthen community outreach, health promotion, social rehabilitation, and referral processes. For secondary care, recommendations included that there be inpatient and outpatient mental health services at all hospitals and strengthened inter-sectoral governance structures to manage MNS services. For tertiary care, recommendations included bolstering services for pediatric and elderly populations, developing initiatives to tackle substance use disorders, and supporting secondary care services with expertise. Further, it encouraged the use of public-private partnerships to innovate the mental health system. Finally, it recommends adequate training, retraining and continuing professional education on screening, identification, and treatment of MNS conditions at all levels of care for key medical personnel [34, 35].

Finding innovative approaches to increase access to effective treatments for MNS disorders in LMICs like Nigeria is in line with WHO Sustainable Development Goals [35]; however, the challenge is to find feasible, acceptable, effective, and sustainable strategies. An effective approach is training non-specialist mental health workers to deliver packages of mental health care under the supervision of mental health specialists in a collaborative, stepped-care, task sharing approach [36]. A common effective strategy for scaling-up mental health care is implementing the Mental Health Gap Action Programme (mhGAP) [37]. This programme is an initiative of the WHO to help national and subnational leaders scale-up mental health care in their communities. Included in this program is the mhGAP-Intervention Guide (mhGAP-IG) that contains a package of interventions for assessing and managing MNS disorders [38]. The main goal is to build and strengthen non-specialists’ capacity in the detection, treatment, and management of MNS conditions through stepped-care, collaborative task sharing. The full modules of mhGAP-IG include: Introduction, Essential Care and Practice, Depression, Psychoses/Mania, Epilepsy, Child and Adolescent Mental and Behavioral Disorders, Dementia, Disorders due to Substance Use, Self-Harm/Suicide, and Other Significant Mental Health Complaints. Although there are no specific anti-stigma modules in the mhGAP, it contains discussions on avoiding stigmatizing language, showing empathy, and increasing awareness about mental illness in the community [38]. There is also evidence to show that stigma improves among trainees pre-and post-mhGAP training [39].

The mhGAP-IG has been implemented and evaluated in many countries. In a review of 162 studies, Keynejad et al. concluded that more research is needed to look at mhGAP implementation especially focused on contextual adaptations [39]. In other state-wide mhGAP projects in Nigeria, only clinical outcomes and trainee skill retention have been evaluated so far [27, 29, 30]. There is need for more studies tailoring training programs and content to local and general care contexts. This can potentially lead to more acceptance and increase the chance for sustainability. By applying a convergent, mixed-methods approach [40], this proof-of-concept study aimed to evaluate the pilot of the mhGAP-IG-based and stigma-focused *Health Action for Psychiatric Problems In Nigeria including Epilepsy and SubstanceS (HAPPINESS)* project. In particular, this study identifies implementation barriers, facilitators, and opportunities, with a focus on the impact of its training component on mental illness stigma among trainees.

### Methods

#### The HAPPINESS Project Intervention

In 2018, the Yale Global Mental Health Program and CBM International, in collaboration with Imo State University Teaching Hospital (IMSUTH) and Imo State Primary Healthcare Development Agency, initiated the HAPPINESS pilot project [41]. During the planning phase of the project, needs assessment meetings with stakeholders took place to prepare for the pilot project’s launch. Stakeholders (e.g., mhGAP-IG experts and PHC staff) convened to discuss the needs of staff and common MNS disorders seen in the community. Together, a team of researchers, psychiatrists, and public health officials adapted and used the mhGAP-IG [38] to train primary healthcare workers (i.e., community health extension workers, nurses, and non-specialist physicians) to assess and treat MNS disorders in their communities while consulting or making referrals to specialists as clinically indicated. There were six main components to the HAPPINESS pilot project: *Training, Refresher Training, Clinical Practice, Support Supervision, Community Engagement, and the Drug Revolving Fund (DRF)*.

#### Training

The training aimed to improve knowledge of MNS disorders, attitudes towards persons with mental illness, and skills in assessing and managing MNS disorders. Of the nine modules in the mhGAP-IG, we chose *Essential Care and Practice, Depression, Psychoses/Mania, Epilepsy, and Substance Use Disorders* and made relevant adaptations based on local needs. We also created a module that focused on stigma reduction. Adaptations, module selection, and the idea to embed a stigma module into the mhGAP were decided during the needs assessment meetings.

The module on *depression* teaches symptoms/signs, differential diagnoses, assessment, including risk assessment, and biopsychosocial treatment planning over time. The module on *psychoses/mania* teaches both psychosis versus mania in bipolar disorder symptoms and signs, diseases’ natural history and course over time, assessment, and treatment planning including managing adverse reactions and side effects of medications. The module on *epilepsy* teaches convulsive versus nonconvulsive seizures, contextual symptoms and signs, causes, and assessment (especially reactive seizing). The module on *substance use disorder (SUDs)* introduces the misuse of substances (alcohol, opioids, benzodiazepines (BZDs), khat, tobacco, stimulants), their signs and symptoms, biopsychosocial impact, assessment of SUDs, and the biopsychosocial treatment and management over time. It also includes motivational interviewing and management of medical and psychiatric emergencies related to SUDs such as intoxication, overdose, and withdrawal. The module on *stigma reduction* discussed common, local stigmatizing language, myths, beliefs, and ways to decrease associated stigma, discrimination, and human rights violations.

The training was delivered in person, facilitated by five trainers experienced in mhGAP-IG training. It was a 9-hour per day, 5-day training (with scheduled breaks) that contained sessions composed of didactics, group workshops, and role-plays. Didactics were based on mhGAP-IG modules with adaptations made, following initial needs assessment meetings with community stake holders and clinicians, to include local content, examples, and narratives (see Table 1). Group workshops were interactive sessions of 5 to 7 people each who read, reviewed and discussed identified topics. Role-plays were enacted by 3 trainees (that took turns as a client, clinician, and observer) and observed by a facilitator. They simulated scenarios such as initial evaluation, psychoeducation, and first aid for seizures. Trainees completed the mhGAP-IG pre-and post-training test before and after each day of training on the specific modules. Lastly, we also included *observed practice sessions* to evaluate trainees on MNS screening, assessment, treatment, and follow-up planning. Post-training certification was based on the following: 100% attendance to all training activities, a score of at least 90% on the post knowledge test, and a “pass” grade on the observed practice session as scored by trainers according to pre-set competency rubrics (patient assessment, patient education, diagnosis, and treatment). A one-day in-person refresher training was scheduled for every six months for all trainees and topic-based discussions using a group WhatsApp forum was planned for every two weeks. Refresher training topics included depression and substance use as these were deemed high priority by clinicians. WhatsApp forums were led by primary care nurse trainees on a rotating basis (nurses from different participating PHCs took turns every two weeks) and topics varied depending on need (e.g., medication management and side-effects, psychosis).

A few adaptations to the training were made in consideration of the local setting as suggested by the primary health care teams. First was the inclusion of specific potential community resources for patients, including local churches, women’s groups, youth clubs and local hospitals, in the relevant modules. Second was the choice of mhGAP-IG modules to train on. This was made based on feedback from the stakeholders and experts during needs assessment about the common disorders they see in the area.

**Table 1:** Examples of adaptations to the mhGAP modules on psychosis, epilepsy, and substance-use disorders

mhGAP Module	Section	Examples of adaptations
Psychosis	Introduction to psychosis	- Identified and listed local names for psychosis/mania: e.g., “Isi ngbaka”, “ara”, Isi nmebi” - Identified and listed local myths and beliefs about psychosis/mania: e.g., “untreatable”, “once the affected persons go to the market, it becomes incurable”
	Assessment of psychosis	- Incorporated local concepts of bizarre behavior; e.g., “ogbanje”, “mami-water”
	Management of psychosis	- Identified and listed local brand names/generic equivalents for antipsychotics/mood stabilizers on the essential drug list: e.g., *Lanzep/Prexal = Olanzapine*
	Role-Play	- Developed script for role play vignette in local *Igbo* language
Epilepsy	Introduction to epilepsy	- Identified local myths and beliefs about seizures: e.*g. It is infectious, it is a spiritual attack, it is untreatable*
	Assessment of epilepsy	- Identified and listed local names for seizures: e.g., *“akwukwu”, “ihe odido”*
	Management of epilepsy	- Identified and listed local brand names for anti-seizure medications on the essential drug list: e.g., *Cartol, Epicar = carbamazepine* and *Epilim Chrono = valproate* - Identified and listed available local specialist/tertiary care centers for referral
	Role Play	- Developed script for role play vignette in local *Igbo* language
Substance-use disorders	Introduction to disorders due to substance use	- Identified, listed local names for commonly used drugs and alcoholic beverages. Example: Liquor like rum, bourbon called “ogogoro”, Cannabis: called “Igbo”, “ahihia” “anwuru ike” and Cigarette and tobacco products called “anwuru”
	Assessment of disorders due to substance use	- Identified and quantified local measures of alcoholic beverages using NIDA guidelines/standards
	Management of disorders due to substance use	- Identified, characterized, and listed available community resources for people with SUD
	Role Play	- Developed script for role play vignette in local *Igbo* language

#### Clinical practice

Informed by the input from community stakeholders, we developed a clinical practice Standard Operating Procedure (SOP) for all the trainees depending on their roles in the primary health center (see Additional file 1). This document streamlines patient healthcare delivery, continuity of care, as well as clinical support and regular program evaluation. For example, the design and development of a personalized pocket-sized appointment reminder was an accessible and affordable way to facilitate treatment adherence and support paper chart medical record organization. The SOP also included a clear flowchart for *Screening, Assessment, Treatment, Follow up, Documentation*, and *Periodic evaluation.*

#### Community engagement

The project team leaders participated in a local radio morning show to raise awareness of the project and provide public health education about mental illness and emotional health. Trained primary health workers also engaged their local communities via local churches, town hall meetings, and visits to traditional rulers. The HAPPINESS project’s social media platforms (Facebook, Instagram, and Twitter) also engaged with local groups, individuals, and organizations to create mental health awareness and disseminate information about the project.

#### Drug Revolving Fund (DRF)

The project utilizes a DRF to ensure consistent availability, affordability, and accessibility of high-quality psychotropic and anticonvulsant medications for those who need it [42]. Medications are dispensed with a marginal mark-up but below market retail price. Any generated net profit is used to offset logistic issues and ensure the sustainability of the scheme (see Additional file 2 for further details).

### Setting

Imo state has a population of about 4 million people, with 527 PHCs staffed by 453 nurses, 76 community health officers, and 864 community health extension workers spread across the State’s urban and rural areas (as of 2017). Each center has a part-time or full-time covering physician. During initial planning meetings, 10 PHCs from five local government areas of the State were selected for the pilot study based on the availability of staff (to take over while some attended training), proximity to the state capital (for physical accessibility), and geopolitical representation.

### Study design and measures

This is a convergent mixed-methods [40] proof-of-concept study that aims to understand the implementation of the HAPPINESS pilot project quantitatively and qualitatively, with a particular focus on stigma. The quantitative questionnaire captures the effect of the HAPPINESS project training’s stigma components on mental illness stigma among trainees. The qualitative interviews explore trainees’ experiences of the project (e.g., project training and support) as well as barriers (e.g., resources availability), facilitators (e.g., aspects of training materials), and opportunities (e.g., future directions) related to the project. These discussions offer insight into project implementation as well as trainees’ perceptions and knowledge regarding mental health, which can be converged with quantitative results on stigma.

#### Questionnaires

As a part of the HAPPINESS project’s SOP, all trainees completed a paper-based pre-and post-initial training stigma questionnaire that captured the local stigmatizing beliefs and negative attitudes about mental disorders. The questionnaire took 15–20 min to complete. Trainees were not additionally compensated for responding to the questionnaire but were given meals during the training and reimbursed for transportation costs. Trainees’ perceptions of mental disorders and attitudes towards people with mental illness were assessed using a 43-item questionnaire (see Table 2) that was created using (1) social distance questions from the Fear and Behavioral Intentions (FABI) Toward the Mentally Ill questionnaire, (2) social stigma, social acceptance and possible treatment options questions from the Community Attitudes to Mental Illness (CAMI) scale, and (3) items about conceptions of mental illness causes from a questionnaire developed for the World Psychiatric Association (WPA) Program to Reduce Stigma and Discrimination [43–45]. These three stigma measures are well-validated and widely used in stigma studies in Nigeria [23]. We have pilot-tested and used this 43-item questionnaire in previous studies in Nigeria. It includes four subscales (acceptance of socializing with people with mental illness, favorable attitudes towards normalized activities and relationships with people with mental illness, beliefs in witchcraft as a cause of mental illness, and endorsement of a biopsychosocial perspective of mental illness) [46–48].

**Table 2:** Stigma Questionnaire Questions (paraphrase) and Subscales

A. Socializing	
I would have a former psychiatric patient as a friend.	
I would live with a next-door neighbor who is a former psychiatric patient.	
I am not afraid of people with mental illnesses.	
I am not afraid of making conversation with people with mental illness.	
I would have conversation with neighbors who previously had mental illness.	
I would invite a previously mentally ill person in my house.	
I would marry a person who was previously mentally ill.	
I am not ashamed if someone in my family was diagnosed with mental illness.	
I am not upset working on the same job with a mentally ill person.	
I would not avoid conversation with a neighbor who is mentally ill.	
B. Normalizing Relationship	
Mental illness is an illness like any other illness.	
The best therapy for mentally ill people is to be a part of society.	
People with mental illness do not tend to be retarded.	
I would be willing to work with somebody with a mental illness.	
People with mental illness are far less of a danger than people think.	
I would maintain a friendship with a person with mental illness.	
Residents should not be afraid of people coming to their neighborhood to receive mental health	
Mentally ill people can work in regular jobs.	
Persons who show signs of mental illness should not be immediately hospitalized.	
Mental illnesses are caused by poverty.	
C. Witchcraft	
Mental illness is not caused by someone putting a curse on you.	
Mental illness is not caused by witchcraft.	
Mental illness is not caused by possession by an evil spirit.	
Mental illness is not caused by God’s punishment.	
Mentally ill people can be treated outside of a hospital.	
D. Biopsychosocial	
Virtually anyone can become mentally ill.	
Mental illness is caused by a brain disease.	
Mental illness is caused by physical abuse.	
Mental illness is caused by biological factors.	
Mentally ill people are not dangerous because of violent behavior.	

A separate paper-based questionnaire was also administered to document self-reported sociodemographic characteristics (age, gender, and years of education), profession (i.e., community health extension worker, community health officers, doctor, or nurse), where they were born, and where they currently live.

#### Qualitative interviews

A purposive sample was recruited by reaching out to stakeholders from the pre-project needs assessment meetings (including primary care doctors, nurses, mental health specialists, state health officials, etc.). Semi-structured interviews were conducted after the initial training and one refresher training between 2019 and 2020. These interviews explored participants’ experience of the project training and pilot implementation, including their perspectives of the project’s impact as well as implementation barriers, facilitators, and opportunities that may exist in the context (e.g., organizational or structural). The interview guide was developed with the Consolidated Framework for Implementation Research (CFIR) with a focus on *“innovation characteristics”*(HAPPINESS project training, materials, and implementation), *“outer setting”* (healthcare system and community perceptions of mental health), *“inner setting”* (primary care clinics structure, hours, and functioning), and “*characteristics of individuals”* (changes in participants’ skills and personal beliefs) [49]. Development of the interview guide was an iterative process including independent review and revision by three researchers and pilot-testing with 3 participants (a nurse, a doctor, and a community health extension worker) to ensure the relevance of the items and areas explored. Participation was voluntary and uncompensated. Each interview ranged from 20–60 minutes using a structured interview guide and additional prompts as needed during the interview for clarifications and relevant details. An overview of the interview guide can be found in Table 3.

**Table 3:** Interview Guide Overview

1. Please give me a brief description of your job and what your average workday looks like? (When do you come in? Where do you spend most of your day? Who do you interact with the most? What takes up most of your time?)	
2. Did you participate in the HAPPINESS project training and refresher training? If so, what are your initial thoughts about the training/refresher training?	
3. Are there any aspects of the training that you think need to be changed (i.e. timing, schedule, duration, content, trainers, etc.)?	
4. How has the training affected your work with patients?	
5. How was your experience with the Drug Revolving Fund? Was it helpful?	
6. What is your perception of the quality of supporting materials (i.e. training modules and other documents)?	
7. In your opinion, how well was the HAPPINESS project integrated into primary care?	
8. What kinds of incentives are there to help ensure that the implementation of the HAPPINESS project is successful?	

### Data analysis

In our convergent mixed-method approach, results from this study’s quantitative and qualitative components were integrated using a narrative, contiguous approach [40]. This involved separately analyzing and reporting findings from each component, followed by merging complementary findings. Specifically, we merged the quantitative component’s measure of changes in stigma among trainees pre-and post-training with qualitative themes and sub-themes that demonstrated attitudes and beliefs among trainees regarding mental illness. It was necessary to merge these results since quantitative measures alone fail to capture nuanced perspectives about stigma. Similarly, asking about informants’ internalized stigma towards people with mental illnesses may be challenging in an interview format alone due to personal biases.

For the questionnaires, thirty questions were included in the analysis and categorized into four subscales as per previous studies that utilized the same questionnaire (*socializing, normalizing, supernatural causation, and biopsychosocial approach*) [46–48]. Responses were analyzed using a paired sample t-test conducted on each of the four subscales using the R statistical software. Variances for each of the subscales were considered unequal except for the biopsychosocial subscale; however, deeming the variance equal or unequal did not affect the results of the t-test.

The qualitative interviews were conducted by NR in person and audio recorded with participants’ consent. Interviews were transcribed by CC with the assistance of NR’s interviewer field notes in cases where audio was difficult to decipher. Thematic analysis was chosen as a way to inductively derive themes from our transcripts [50]. This was chosen as opposed to deductive coding using the CFIR framework because although our semi-structured interview guide directly asked about project-specific aspects, we wanted an analytical process that would allow us to flexibly find patterns within the data about any contextual barriers and facilitators related to the project, for example, the project training, clinical practice guidelines, community engagement or the DRF. Coding was done by CC using the Dedoose software (Version 8.3.17), where transcripts were uploaded. The initial round of coding was descriptive and hierarchical, where initial parent and child codes were coded. Throughout this process, initial codes were collapsed or re-labeled on Dedoose where necessary. These codes were defined to form an initial codebook and the codebook was re-applied to transcripts until saturation was achieved (no emergent codes). This codebook was downloaded from Dedoose as a Microsoft Word document (where codes and corresponding transcripts were automatically listed) and collaboratively reviewed by CC, TI, and KW to ensure clarity and to extract themes and sub-themes. After the final codebook and themes were formed, transcriptions that corresponded to each theme and sub-theme were analyzed to generate findings and conclusions regarding the HAPPINESS pilot project and training.

## Results

### Questionnaires

Table 4 summarizes the demographics for the initial respondents (n = 34) and the subsample of respondents who completed both pre-and post-training questionnaires (i.e., the analyzed sample; n = 13). The average age of the initial sample was approximately 45 years old (*SD* = *7.73*) and approximately 43 years old (*SD* = *8.74*) for the analyzed sample. The average years of education were approximately 19 years (*SD* = *3.77*) for the initial sample and approximately 18 years (*SD* = *2.68*) for the analyzed sample.

There were no significant differences between initial respondents and incomplete or non-respondents. The initial respondents and the analyzed sample were predominantly female (85% and 92%, respectively). The initial respondents were mostly doctors, then community health extension workers (CHEWs), then nurses (53%, 35%, and 12%, respectively). The analyzed sample was also predominantly doctors, then CHEWs, then nurses (62%, 31%, and 8%, respectively). Most people were born in rural areas (79% for the initial respondents, 92% for the analyzed sample), and none were born in semi-urban areas. For the initial respondents, 39% were currently living in a rural area, 33% in an urban area, and 27% in a semi-urban area. For the analyzed sample, 23% were currently living in a rural area, 38% in an urban area, and 38% in a semi-urban area. Thus, our analyzed sample of 13 people was less rural living than the initial respondents of 34 people.

**Table 4:** Demographic Information

		Initial Respondents (*n* = 34)		Analyzed Sample (*n* = 13)	
	Average Age	**45.09** (7.73)		**43.08** (8.74)	
	Average Years of Education	**18.94** (3.77)		**17.77** (2.68)	
		**n**	**%**	**n**	**%**
Gender	Male	5	15%	1	8%
	Female	28	85%	12	92%
Job	Community Health Extension Worker	12	35%	4	31%
	Community Health Worker	0	0%	0	0%
	Doctor	18	53%	8	62%
	Nurse	4	12%	1	8%
Born	Urban	7	21%	1	8%
	Rural	26	79%	12	92%
	Semi-urban	0	0%	0	0%
Currently Live	Urban	11	33%	5	38%
	Rural	13	39%	3	23%
	Semi-urban	9	27%	5	38%

Demographic information was collected from the self-report stigma questionnaire for the original set of respondents (*n* = 34) and the final sample (*n* = 13) included in the analysis. For average age and years of education, the numbers in parentheses are the standard deviations. One initial respondent did not provide their gender, place of birth, and place they currently live

Table 5 shows the results from the paired two-sample t-test. Overall, there were fewer stigmatizing attitudes and beliefs towards people with a mental illness after the training compared to before. This is demonstrated by the mean responses of each subscale (pre versus post) but also by the t-test results. Scores significantly improved on three of the subscales (socializing, normalizing, and causation). Post-training, respondents reported more acceptance of socializing with people with a mental illness, *t*(12) = 3.07, *p* = 0.01, more favorable attitudes towards normalized activities and relationships with people with a mental illness, *t*(12) = 5.03, *p* = 0.0003, and less of a belief that witchcraft was a cause of mental illness, *t*(12) = -2.55, *p* = 0.025. There was no significant effect of the training on the biopsychosocial subscale, *t*(12) = 0.74, *p* = 0.472.

**Table 5:** Change in Attitudes and Beliefs of People with Mental Illness

Subscale	Pre-Training Mean	Post-Training Mean	T-Value	P-Value
Socializing	1.72 (0.24)	1.88 (0.10)	3.07	0.010
Normalizing	1.55 (0.18)	1.79 (0.10)	5.03	0.0003
Witchcraft	1.37 (0.34)	1.12 (0.10)	-2.55	0.025
Biopsychosocial	1.58 (0.21)	1.69 (0.16)	0.74	0.472

Subscale means are based on an answer of 1 for disagree and 2 for agree to questionnaire statements. For the socializing subscale, 1 indicates less acceptance of socializing with people with a mental illness (and 2 indicates more acceptance). For the normalizing scale, 1 indicates less favorable attitudes towards normalized activities and relationships with people with mental illness (and 2 indicates more favorable). For the witchcraft subscale, an answer of 1 indicates a belief that witchcraft does not cause mental illness (and 2 indicates a belief that it does). Numbers in the parentheses are the standard deviations

### Interviews

A total of 11 semi-structured key-informant interviews were conducted. These included 4 state health officials (from various organizations in Imo State; all doctors themselves) and 7 HAPPINESS project trainees, comprising of 4 nurses, 1 primary care doctor, and 2 mental health specialists (1 clinical psychologist and 1 psychiatrist). The interviews highlighted respondents’ perspectives on specific aspects of the HAPPINESS pilot project, but also, more generally, existing barriers within the context of health care delivery in Imo State. A summary of these findings is presented in Table 6. Some interviewees’ specific job titles are omitted to preserve anonymity (as there are few of these roles in Imo State). Primary care nurses and doctors will be noted as *primary care workers*. Psychiatrists and psychologists will be referred to as *mental health specialists*.

**Table 6:** Themes from the qualitative interviews with key stakeholders of the HAPPINESS project

Theme	Sub-Themes	Summary
HAPPINESS project impact	new skills	Newly gained or improved ability to advocate for patients and detect, diagnose and treat mental illness.
	ideological changes	An improved awareness of mental illness, leading to more empathy and respect towards patients.
	drug revolving fund	An overall positive impact on drug access.
Contextual threats to address	lack of awareness	High levels of misinformation and stigma in the population leading to undetected mental illness.
	physical/structural/systemic barriers	Poor access to mental health care (road access and availability of specialists), lack of funding, and lack of basic healthcare tools (some, unrelated to mental health).
Project-specific remarks and opportunities	promoting early detection and raising awareness	Educating community members and families to bring patients to a primary care center instead of alternative types of treatment (e.g., religious).
	supervision	Lack of supervision on a day-to-day basis for trainees.
	training structure & components	Adding additional topics, increasing the length of training, and tailoring training to each type of health workers’ needs.
	trainee recruitment & retention	Providing trainees additional stipends and recruiting trainees who are more deeply motivated to expand their knowledge on mental health care.
	involving more people	Training more people and building more partnerships with local organizations.

#### The HAPPINESS Project’s Impact

##### New Skills

Interviewees cited many changes to the way trainees took care of patients after the training workshop:

“*Before the training, I was referring my patients with MNS conditions. But after the training, I am now able to diagnose and treat my patients. Like, treating [those that present] with psychosis. After treating them, they become better and go back to their families, they gained back their responsibilities.“ – Primary Care worker*.

Many noted a drastic improvement in their ability to detect, diagnose, and treat mental illness. For instance, the workshop taught them how to record and use Patient Health Questionnaire-9 (PHQ-9) and Brief Psychiatric Rating Scale (BPRS) scores to screen for and assess the severity of common mental disorders. Trainees also reported increased belief in the importance of counseling:

*“I think the most important thing is counseling. Every patient needs counseling. Because one out of every 10 patients have an MNS condition, even silently […] so we counsel them […] they tend to open up things that you may not have really known. Or they have an issue that they never wanted to say. We need to create that good relationship with the patient and try to let them know that everything I discuss with you is private, is between you and (inaudible), except when they give you consent to discuss these things [with others]” – Primary Care Worker*.

A psychiatric nurse also noted marked improvements in her ability to diagnose and treat patients with a mental illness (including providing the correct drug dosage), despite being a psychiatric nurse who was already formally trained in psychiatry:

*“We [found] it difficult before, to give diagnosis but after the training, most of us, even those who are not psychiatric nurses can now identify the mentally ill…we now make the right diagnosis. Even when I can’t do it myself, I’ll call (the mental health specialist) and [they’ll] give the right diagnosis. We give the right diagnosis, we give the right drug […] with this project, it’s actually really improved my knowledge and gave me [a] wider knowledge of psychiatry, so I’m impressed and happy”*.

*“Some of them [used to] react to the dosage but nowadays, since this training, none of my patients have reacted to dosage, because I give them the right drug dosage now”*.

Lastly, many respondents indicated feeling confident to apply what they learned in the training workshop to practice. Notably, there was one occasion where the psychiatric nurse advocated for their patient, who would have been arrested by the police if they did not step in:

“*he had a problem with somebody and they started beating him, the police arrested and they locked him up, so I went to the police station and I told the cops in charge, I said, please don’t beat this person, he is a mentally ill patient, he doesn’t know what he’s doing… he’s not mentally sound, this and that, and I said he’s my patient [… ] I stood my ground because I know what I am saying…”*.

The training equipped the nurse with the confidence to advocate for their patient, which was rooted in their new and more expansive knowledge of mental illnesses.

##### Ideological Changes

The training also led to a changed attitude regarding mental illness. Many developed a sense of empathy and respect for patients. This went on to change the way they interacted with patients, as well as how they provided treatment to patients:

“… *most of them need privacy [for the] patient doctor-relationship, [we need] to create that perfect environment for them … so I ask, are you okay? Do you need somebody to stay with us? […] I’ll keep the patient’s relative outside … just to make the patient feel comforted. And first off, I need to let them know that whatever we’re going to discuss, [will] be confidential, [will] be between me and the patient” – Primary Care Worker*.

*“I’ll go ahead to ask them if they permit me to bring in a colleague or a nurse […] and if they say no, I don’t go ahead, I respect their decision” – Primary Care Worker*.

These primary care workers described how they altered the environment and treatment approach to make patients feel more comfortable and that their autonomy was being respected. They also noted pre-workshop versus post-workshop ideas about people with MNS disorders in the following quote:

*“[I] felt that [before], [we were] not really tending to MNS […] because of, probably because of their environment and how violent they can be. You know? But after that training, I was able to […] show love to these patients. They can be understanding to you, can listen to you or […] instructions […], the training made me realize that we are all one […] they have the same problem […] it’s just a sickness that needs to be treated” – Primary Care Worker*.

This mentality of “they are just like us” and that the patient has an illness that needs to be treated is evidence that some respondents may have developed a biopsychosocial view of mental illness. Related to this mindset, some participants also mentioned the importance of helping patients integrate back into society and regular life (e.g., their workplace) after treatment.

##### Drug Revolving Fund (DRF)

In general, respondents had positive views of the DRF. They noted that although it involved a lot of “paperwork”, it led to drastic improvements in the ability of patients to pay for and adhere to treatment. The following quotes show, from multiple perspectives, that the DRF is beneficial for patients and affordable:

*“[…] financial constraint is another problem because you have a family that doesn’t have money to afford medications […] so if you have no money, you can’t be treated. So, I think the program can help to either reduce the funding from bill of these patients or trying to subsidize so everyone can access treatment irrespective of your income”- Primary Care Worker*.

*“They don’t complain even though financially, things are not suitable for most people even though it is very, very affordable for them, but some of them take it with ease, some come and pay half and half. But in my clinic […] I told them don’t ever stay at home because you don’t have money to take your drugs. Please take your drugs, please be stable. By the time your patient is stable and improving, the patients’ relatives will not hesitate to pay for the drugs […] they pay for it” – Psychiatric Nurse*.

The main limitation of the DRF is that it only covers oral medications that are found on the country’s Essential Drug List [51]. This removes part of the cost barrier to accessing treatment, but it is still limiting the types of conditions that primary care workers can handle. Additionally, there are also situations where health workers must administer a drug via *intramuscular injection* as opposed to *oral administration*; for example, when health workers encounter severely agitated patients:

“I’m *using the drug revolving fund. But the problem with that, [we don’t] get access to injectables, we only have oral drugs. And like…Yes, most psychotic patients find it hard to take oral drugs initially. If you can inject them, they will calm down and then start taking oral drugs” – Primary Care Worker*.

#### Contextual Threats to Address

All the providers we interviewed noted many existing barriers to mental health care in Imo State that could influence the success of the HAPPINESS project. The following quote by a mental health specialist highlights the main barriers that are experienced by people who are seeking care:

*“There is no insurance to cover mental health in the country, so treatment is basically out of pocket. The barriers to care [include] the distance [and] accessibility to care. Most of the patients live in the rural areas, [but] the facilities are located in the urban areas. So, there is a very large distance for them to [travel] to access care, which is very expensive. [Other barriers include] […] lack of knowledge and lack of awareness about mental health. A lot of people still believe that they are caused by spiritual power, and the severe lack of… nurses, doctors, social welfare workers, therapists, I don’t think there’s any in the state now, clinical psychologists are very few…”*.

##### Physical/Structural/Systemic Barriers

An inadequate physical environment, lack of funding, and poor availability of mental health professionals were noted as barriers to mental health care that have persisted in Imo State.

Many respondents noted a lack of basic health care tools in many clinics (e.g., weight and height scales). Moreover, project supervisors claimed that routes leading to PHCs were difficult to physically access.

Lack of funding was commonly mentioned as a barrier to seeing a mental health worker or to acquiring medications that are prescribed for treatment. There are very few mental health specialists in Imo State; thus, people living in more rural areas have difficulty accessing treatment. Moreover, people must be screened and referred before they can access care from a mental health specialist (they may not self-refer).

Regarding the supply of mental health workers, there are relatively few programs in Nigeria that train and produce psychiatrists:

*“We have two colleges, the [National Postgraduate Medical College of Nigeria] and the [West African College of Physicians] for training of a specialist. And then when you graduate, the training averages 4–6 year for you to become a fellow of the college and when you graduate you become a psychiatrist, a general psychiatrist […] because of brain drain, most of the personnel (these trained psychiatrists) are outside of the country”- Mental Health Specialist*.

Aside from the limited availability of mental health specialists, there are a limited number of health workers (potential trainees for the HAPPINESS project) that are already overburdened. Many trainees noted working full days, 7 days a week, and frequently working overtime:

*“[I work] from 8 o’clock. The official time is [to] 4 o’clock, but I go beyond that if I have a patient or if [anyone] calls me on the phone that they’re coming, I have to wait for them. So specifically, I don’t have times when I don’t see anybody and leave. – Psychiatric Nurse*.

##### Lack of Awareness

A severe lack of mental health awareness was frequently noted by all the interviewees as a common upstream challenge to receiving adequate care and early diagnosis. In particular, they noted high levels of misinformation and stigma among the general population and local community leaders. The following quote by a primary care worker details the severity of stigma that people are facing:

*“Most of them are scared. They don’t want to tell their neighbors, or [their] son or [their] daughter or [their] friend […] that they are having this problem, so that they don’t stigmatize them. So, they tend to hide this problem, and this problem is killing the patients. So, it’s a very big problem, so I think that has been a stumbling block”*.

Another theme related to a lack of awareness is that underlying mental illnesses are often ignored and go undetected because mental health is the last priority. At the health system level, resources and attention tend to go towards acute and chronic physical illnesses and conditions, rather than mental health. This lack of awareness leads to a delay in receiving the correct and suitable care. One state health official demonstrates this in the following quote:

*“The person that comes with the thing that is malaria, that’s the reason why they came to the health center. Or they may have typhoid fever, meanwhile the issue [that remains] is depression”*.

#### Project-Specific Amendments and Opportunities

##### Promoting Early Detection and Raising Awareness

The majority of respondents talked about the urgency of taking measures to ensure that people with MNS disorders are diagnosed and treated as early as possible. Many were concerned that those affected by mental illness get exposed to alternative treatments (notably, at churches), which are viewed as improper treatment. These treatments reportedly often involve violence and are perceived as very harmful to individuals:

*“I also experienced a case where this patient had this condition and the parents took the patient to somewhere where they were flogged […] in general in rural [settings], creating awareness that once someone, your child or your daughter, or your son is having this problem, or someone you’re familiar with is having this problem, please don’t do this. You can’t […] look at [the] difference, there are fully steps you can take to ensure that this patient gets treated.“ – Primary Care Worker*.

Many respondents viewed increasing awareness as a key element to early detection and proper treatment. They also believe that raising awareness and educating the public is essential to combating stigma as a barrier to treatment. They suggested raising awareness through paper advertising (e.g., flyers and brochures), media advertising (e.g., TV and radio), and directly engaging with churches. The content of awareness initiatives should include basic information about mental illness and advocate for bringing those (who may have a mental illness) to clinics rather than churches. Additionally, they emphasized partnering with local government leaders on awareness-raising initiatives.

The target for awareness-raising should be the entire community, people with a mental illness, and their families. Specifically, these should focus on educating people on what mental illness is, its treatability and what are safe treatment settings:

*“it also made me to realize that whenever I see families, [I tell these] families – don’t chain [your] loved ones because of these conditions […] I need to intervene as quickly as possible, you know, let them know that this is not the right way to do it” – Primary Care Worker*.

*“probably tell the villagers […] you should accept them, be friendly with them … give them a chance to say something, contribute […] And probably to teach them the signs [to] notice earlier. Because a mentally ill patient may not know because some of them lack insight. Even when you tell them, they say no. So, when they see such people…they bring them to the clinic and they better. They shouldn’t be hiding them because of stigma or what people will say, they should help the patients” – Psychiatric Nurse*.

The person who has a mental illness may not realize they have a mental illness; thus, it is up to the community and people around them to not impose and exacerbate stigma, to recognize that someone may have a mental illness, and should consider seeking professional help. Moreover, a reduction of stigma in the communities will be conducive to helping individuals recover.

##### Supervision

In-person supervision post-training was also key to project implementation:

“*[After the HAPPINESS project] I have someone around that always visits [the] office. I feel happy when [the project supervisor] around, if I have patients I will say, talk to them, take care of them. And [the project supervisor] does” – Psychiatric Nurse*.

However, supervisors noted that they are strained and have difficulty accessing the primary care centers:

*“We do supervision, the circuits. Because we need to supervise them. Not just training them and leaving them, they need to be supervised […] I’m the only clinician at the training part of the training now. Though we have a project coordinator who goes twice a month to collect data.“ – Mental Health Specialist*.

*“We need to supervise them. Not just training them and leaving them, they need to be supervised. So, it’s been difficult doing the supervision because of the state of the routes, the routes are very bad. We don’t have a project vehicle, which is a very urgent need. The roads and centers cannot be accessed using a normal [vehicle]” – Mental Health Specialist*.

##### Training Structure and Components

Respondents commented favorably about the training materials (i.e., the adapted mhGAP training materials) and found them to be a helpful resource when they are unsure about a patient or unable to reach a mental health specialist. Moreover, the WhatsApp forum was transformative, especially when there were no mental health specialists physically or virtually available. It provided a quick way for trainees to ask for help and seek advice from specialists and fellow trainees.

Participants also suggested several improvements to the training itself, including additional training materials and topics, and amending the training structure and length. For additional materials, one respondent suggested making a website to collect information from the training:

“…*this program has been so helpful, but I still want to know more. You should create a platform, we already have one (the WhatsApp forum), but I don’t know if we have everyone. We should have access to a website where we can see materials to improve our knowledge about MNS conditions that would be great” – Primary Care Worker*.

Some respondents also desired new topics such as child mental disorders, cognitive behavioral therapy, and other neurological disorders. Some respondents also reported that more in-depth training would help them differentiate between different disorders and improve identification and diagnosis (since many mental illnesses have similar signs and symptoms).

*“MNS conditions can be confusing. So, it can really be confusing, and we need to understand every aspect of these conditions. I think they need to go deeper. So, we can be able to differentiate. Um, these conditions, everybody sees it, then most of the times it can look like the symptoms” – Primary Care Worker*.

*“I want to know the difference between depression and anxiety, and manic depression. I want to know the differences. Manic depression and depression and anxiety” – Psychiatric Nurse*.

Most respondents believed that the training was “rushed” and that there was “too much information”, but they liked the way materials were taught (e.g., using role-play). For this reason, many suggested a 7-day training, with a refresher every 3 months (compared to current 5-day training with a refresher every 6 months). Respondents also indicated that the project should continue to provide travel stipends and lodging for the duration of the training:

*“…residential training is good, and you know the cost. They have to take care of the logistics and also pay for the transports and all the other out of pocket expenses” – State Health Official*.

*“…if you increase it to 7 days, of course, it would be better, but it needs to be an in-house training so that some of the rural health centers [can attend]” – State Health Official*.

Some respondents also expressed that some trainees felt “rushed” because they were coming in with different expertise. Thus, many suggested training different professions separately:

*“So I think the training can be increased, you can have a separate training for doctors and nurses, then you have for the CHEWs, you also have a separate training for them because for those you need to be slow […] some of the CHEWs, they’re going to start from the basics, so they need time” – State Health Official*.

*“I think that doctors should be trained differently, separate from nurses and also CHEWs because this one uses SOP’s (standard operating procedures) that doctors that use to treat. So, the content really shouldn’t be the same” – State Health Official*.

Professionals from different backgrounds may learn at different speeds, and these differences could be better addressed by training them separately. Additionally, each profession has a different SOP, meaning they have different skills and scope of practice for treating patients. Conversely, however, one primary care worker expressed their desire to share information between different types of professions.

##### Trainee Recruitment & Retention

Respondents were favorable towards the current HAPPINESS project retention strategy of only recruiting trainees who will be staying in their location for at least two years. Respondents suggested multiple retention strategies including that the HAPPINESS project should provide travel and lodging for trainees and have adequate resources within clinics. Many respondents also thought it was important that training should only be for those who are motivated and passionate about mental health. Respondents believe that people without motivation will be passive learners and not gain a lot from the training. There was also concern that people will misrepresent their roles after being trained as being a mental health specialist:

*“Somebody can just try this training and put on his or her bag and walk into the village and say, you know, now I am a psychiatric doctor, if anybody, please come to this address. Those are the things you people have to avoid and you use those within the health system. That’s my own advice, it’s an opinion. If you use people that are not into health, they can’t do what I’m doing, in the villages […] with formal training, yes, doctors, nurses, community health workers, yes” – Psychiatric Nurse*.

##### Involving More People

When asked about how the HAPPINESS project could expand, respondents suggested recruiting more trainees and partners.

All respondents agreed that there should be more trainees in the future. This could involve an expansion beyond the 5 local government areas (LGAs), to the rest of the total 27 LGAs, or the inclusion of trainees from a wider range of backgrounds (e.g., village health workers):

*“I still see those 5 local governments, meanwhile we have 27 local governments. So, the training can [be increased] so that all 27 local governments can benefit” – Mental Health Specialist*.

Respondents also suggested forming more partnerships with potential local funders, the local community, schools, churches, *traditional rulers*, and the local governments. They believed that with more links to these community stakeholders, the chances of helping people with MNS disorders increases. *Traditional rulers* (usually selected or appointed with no term limits) were commonly noted as they are the stable, sustainable interface between LGA’s and the communities. Thus, they would be useful in getting community buy-in and long-term support for the project.

### Merged results

We merged data from the stigma questionnaire with qualitative sub-themes that demonstrated a change in participants’ perceptions post-training (either through explicit mention of personal ideological changes or opportunities for the HAPPINESS project to improve). Significant findings from the stigma questionnaire related to findings under the ***ideological changes*** and ***promoting early detection and raising awareness*** sub-themes. The questionnaire found that trainees were significantly improved on the *socializing*, *normalizing*, and *supernatural causation* subscales, which complements respondents’ reports that they developed a better understanding of mental illness and had more empathetic and respectful interactions with patients. These findings were also bolstered by participants’ expressing that the HAPPINESS project should raise more awareness about mental health to prevent alternative treatments (e.g., in churches). The quantitative result for the *biopsychosocial* subscale (the understanding of the biopsychosocial model of causation) was the only subscale with an improved but non-significant change pre-and post-training. However, evidence of improvement in this biopsychosocial view of mental illness was also seen in the ***ideological changes*** sub-theme from the qualitative data, especially where participants acknowledged that people with a mental illness are “just like us.”

## Discussion

This study evaluates the impact of the HAPPINESS pilot project mhGAP-IG training on trainees’ mental illness stigma perceptions and knowledge as well as barriers, facilitators, and opportunities related to project implementation. Findings from the stigma questionnaire were congruent with the qualitative data that showed a favorable change in attitudes regarding mental illness following the HAPPINESS project training. As indicated by the questionnaire, trainees reported significantly more acceptance of socializing with people with mental illness and more favorable attitudes towards normalized activities and relationships with people with mental illness; further, they were less likely to endorse supernatural causes of mental illness. These preliminary quantitative results were supported by emergent themes from the interviews, which showed that trainees gained a better understanding of mental illness, learned new skills in providing diagnosis and treatment, and developed (and acted with) more empathy and respect towards patients. A demonstrated increase in empathy and respect from the interviews also demonstrated biopsychosocial perceptions of mental illness (which was insignificant in the analysis of the stigma questionnaire). Moreover, participants emphasized the importance of integrating patients back into society and regular life (e.g., their workplace) after treatment. This is a key component of the “recovery approach”, which has long been used to guide the creation and delivery of mental health services [52]. The improved knowledge and perspectives regarding stigma among trainees, which were a main focus of our study, are in line with qualitative and quantitative evidence from other LMICs on the impact of mhGAP-IG training [53–55].

A notable concept that emerged from the qualitative component of this study is the negative downstream impact of the lack of public awareness regarding mental illness on people receiving timely and suitable care (particularly seen in our ***lack of awareness*** and ***promoting early detection and raising awareness*** sub-themes). These discussions provide interesting insight into and demonstrate trainees’ understanding of the mental health context of the HAPPINESS project, especially regarding public stigma. Informants often described how lack of awareness among people, their families, and the community-led to delayed care-seeking and detection of a mental illness, which leads to poor management of mental illness. Specifically, they reported that misinformation and stigmatization often alienated people with a mental illness (which may exacerbate their illness) and led them to be brought to churches for treatment (i.e., from the belief that mental illness is caused by spirits). This supports the need for the HAPPINESS project and other mental health programs to organize awareness programs in the community to improve treatment receptiveness. Eaton and Agomoh show that such a program can help increase help seeking for mental disorders in Nigeria, especially if it ties in the availability of mental health services [56].

Regarding the HAPPINESS project training, many respondents recommended increasing the length of time (to have more time to learn) and tailoring training for different health professions (since some clinical workers have had less exposure to the workshop content previously). Additionally, respondents still desired extra training on how to differentiate between illnesses and additional topics they wanted to be covered. Thus, a challenge will be to slow down the training’s pace while adding additional topics. The HAPPINESS project’s refresher trainings can address this need for continuous training; however, longer trainings with new topics may not be financially feasible long-term or accessible to all trainees. Thus, it is crucial to bolster and continue offering alternative learning avenues, such as the HAPPINESS project’s WhatsApp forum and specialist supervision. Ongoing specialist supervision and collaborative and multi-disciplinary peer-to-peer learning (the “Community of Practice” approach) was noted by Fargeh et al. as a solution to similar learning gaps found in initial mhGAP training in Chad, Ethiopia, Nigeria, Guinea, and Haiti [57].

Beyond the training, respondents suggested working closely with community leaders and creating promotional material to improve awareness of mental illness to promote primary care center visits for people who may have a mental illness. The HAPPINESS project had already taken steps to address these avenues via raising awareness on social media and on local radio shows (whose effects may not have been captured at the time of this study). Additionally, some project sites have made outreach visits to traditional rulers, village committees, and councils (with anecdotally positive results so far). This also aligns with recommended best practices for community engagement in global health implementation research and projects [58]. Another issue that the HAPPINESS project has been addressing includes implementing trainee retention strategies. The project team is currently working with the local university to develop this training into a university-based certificate program. This way, trainees will get recognition for the time and effort they spent on building new skills in mental health.

This study adds to the limited literature on the impact of mhGAP-based training on stigma, specifically, how embedding anti-stigma components into mhGAP training can improve mental illness stigma among primary care trainees. The barriers that were identified in the implementation of this project are also consistent with other cultural and contextual challenges to the mhGAP-IG implementation identified by Fargeh et al., including the local perception of mental disorders, the healthcare system, available support for trainees, prior knowledge of trainees, trainee recruitment, and the larger socio-political context [57].

The present study complements previous Nigerian mhGAP-IG projects’ studies that focus mainly on trainee skill retention and clinical outcomes by detailing challenges that arise in initiating a mhGAP-IG implementation project, as well as how external factors (e.g., awareness) contribute to the success of the project [27, 29, 30, 59]. The quantitative component revealed a reduction of stigma among trainees, complemented by qualitative findings regarding trainees’ new perceptions and knowledge about stigma after training. The qualitative component also identified implementation barriers, facilitators, and opportunities for this pilot project which are helpful not only for the growth of the HAPPINESS project but also for other project teams who are initiating or expanding their mhGAP-IG interventions.

Although one of the main strengths of the HAPPINESS project is its use of the widely available primary healthcare system in Imo State, it should be acknowledged that there are challenges to access that go beyond an easily accessible PHC. Most of these factors are connected to the community and system-level stigma and negative attitudes toward mental health, leading to low interest and minimal investment in mental health services. Firstly, this study revealed that a lack of human resources limits aspects of project implementation and day-to-day primary healthcare center functions. Respondents expressed that the lack of psychiatry training programs within Imo State means that local people will unlikely become psychiatrists. With brain drain being very common, Imo State natives that are trained elsewhere may also choose not to return home to practice [60]. Having a small supply of healthcare workers limits the pool of potential trainees and supervisors and the availability of current trainees and supervisors. This may not be a current concern, but as the project expands, more supervisors will be needed to offer online and in-person support. Secondly, there is poor access to adequate healthcare resources, including difficult physical access to primary care centers (mentioned by a respondent as a barrier for supervision) and a lack of basic health assessment tools (e.g., stethoscopes and blood pressure cuffs). Beyond this study, it has been found that primary healthcare facilities in Imo State are often dilapidated, poorly staffed, lack essential drugs, have long wait times, and have high costs for treatments [61, 62]. To combat some of these issues, the HAPPINESS project has given basic medical tools to the partnering clinics. However, there are still limitations to what the project can ameliorate. For instance, although the DRF was highly regarded by the respondents of this study, it only provides oral medications on the national essential drugs list (thus, restricting the types of therapies available) [51].

Beyond challenges within PHCs, there are also severe disparities with regard to individuals’ abilities to pay for care in Nigeria. Even with the establishment of the federal National Health Insurance Scheme (NHIS) in 2005, more than 90% of the national population (and 5 million people in Imo State) remain uninsured [63, 64]. NHIS is also more challenging to obtain for those who are unemployed (which is common for those with a mental illness). In southeast Nigeria (where Imo State is located), 27% of households incur catastrophic health expenditures, and this rate was higher for rural regions and for people with a mental illness (factoring in both direct and indirect costs of the illness) [65–67]. Even if people obtained NHIS, these plans only cover care at certain federal hospitals and do not cover drugs that are not on the essential drug list of Nigeria, which excludes many common psychotropic medicines (e.g., SSRIs) [51]. NHIS plans also do not cover care provided by clinical psychologists, social workers, and occupational therapists, and they also do not cover mental health services such as psychotherapy and addiction clinics [24].

Instead of being fully reliant on federal health insurance programs, the Imo State Health Insurance Agency, in 2019, started to collaborate with the WHO and different unions and health organizations in the community to improve health insurance for people in Imo State [64]. Looking ahead, these improvements to health coverage in Imo State will be highly essential to the success of the HAPPINESS project, as it will increase the number of people who can access PHC services without paying out-of-pocket. Additionally, any upcoming improvements to bolstering primary healthcare’s infrastructure and human resources will be conducive to the HAPPINESS project’s expansion.

There were methodological limitations to this study worth noting. Firstly, due to financial constraints, this was a small pilot, proof-of-concept study that focused on integrating mental health into primary healthcare using the mhGAP-IG in select LGAs in one state in Nigeria. Because of these constraints, it was not possible to train a large cohort of staff (which would have increased survey participants) nor was it feasible to incentivize interview participation, leading to a low sample number. Thus, the findings may not be generalizable to larger portions of the state or the country. Optimistically, the project team has received funding to use the data and lessons learned from this pilot study to conduct a larger, prospective study to evaluate the feasibility, acceptability, and effectiveness of the HAPPINESS intervention across the entire state (Imo State).

Secondly, this study was not designed to evaluate the ***community engagement*** components of the HAPPINESS project as these effects are more challenging to capture within our time frame and with a small sample size of informants that were already actively involved with the project. Future evaluations of the HAPPINESS intervention will aim to capture community perspectives to evaluate this component.

Thirdly, less than half of the questionnaires were complete and included in the analysis, limiting the robustness of the findings. However, the results align with existing literature and provide useful insight into approaches for integrating anti-stigma components into mental health capacity-building interventions in primary care settings. Additionally, the use of convergent data was helpful. For example, our qualitative data found positive ideological changes in trainees’ perceptions of mental illness, which bolstered findings from the quantitative questionnaire. We will incorporate lessons learned in this pilot project to ensure a higher survey completion rate in future studies.

Lastly, this study only examined the perspectives of healthcare workers (doctors and nurses) and health systems leaders. It did not include any community health workers (CHWs), community health extension workers (CHEWs), or the patients’ perspectives. In the next, planned larger implementation study of the HAPPINESS intervention, the study team intends to include perspectives from CHWs, CHEWs, patients, families, and other relevant community stakeholders.

## Conclusions

This study adds to the available evidence on the implementation of mhGAP-IG, a model of care that uses task sharing to integrate mental health treatment into primary care, in Nigeria. It quantitatively and qualitatively evaluates how this model of mental health care can impact perceptions and knowledge of stigma among trainees. It also highlights the barriers, facilitators, and opportunities necessary to consider for this project to improve and grow in the Nigerian context. Future efforts should focus on clinical support, supervision, and health outcomes as well as scaling up and assessing the cost-effectiveness of the HAPPINESS project intervention.

## Supplementary Information

**Additional file 1.** The HAPPINESS Project Standard Operating Procedure (SOP). SOP for clinical record-keeping and reporting for primary care nurses and community health workers.

**Additional file 2. **The HAPPINESS Project Drug Revolving Fund (DRF). Outlines the project’s DRF scheme.

## Data Availability

The datasets used and/or analysed during the current study are available from the corresponding author on reasonable request.

## Abbreviations

HAPPINESS: Health Action for Psychiatric Problems In Nigeria including Epilepsy and SubstanceS
MNS: Mental, Neurological, and Substance Abuse
mhGAP-IG: Mental Health Gap Action Programme–Implementation Guide
CHWs: Community Health Workers
CHEWs: Community Health Extension Workers
DRF: Drug Revolving Fund

## Declarations

All participants provided verbal consent to participate.

## References

[1] Patel V, Chisholm D, Parikh R, Charlson FJ, Degenhardt L, Dua T, et al Global Priorities for Addressing the Burden of Mental, Neurological, and Substance Use Disorders. In: Patel V, Chisholm D, Dua T, Laxminarayan R, Medina-Mora ME, editors. Mental, Neurological, and Substance Use Disorders: Disease Control Priorities, Third Edition (Volume 4) [Internet]. Washington (DC): The International Bank for Reconstruction and Development / The World Bank; 2016 [cited 2020 Jul 24]. Available from: http://www.ncbi.nlm.nih.gov/books/NBK361949/.

[2] Petersen I, Marais D, Abdulmalik J, Ahuja S, Alem A, Chisholm D (2017). Strengthening mental health system governance in six low- and middle-income countries in Africa and South Asia: challenges, needs and potential strategies. Health Policy Plan Oxford Academic.

[3] World Bank. Data for Nigeria, Lower middle income | Data [Internet]. [cited 2020 Jul 11]. Available from: https://data.worldbank.org/?locations=NG-XN.

[4] World Health Organization. Depression and Other Common Mental Disorders - Global Health Estimates [Internet]. Geneva; 2017. Available from: https://apps.who.int/iris/bitstream/handle/10665/254610/WHO-MSD-MER-2017.2-eng.pdf.

[5] Gureje O, Lasebikan VO, Kola L, Makanjuola VA (2006). Lifetime and 12-month prevalence of mental disorders in the Nigerian Survey of Mental Health and Well-Being. Br J Psychiatry.

[6] Aguocha CM, Uwakwe RU, Duru CB, Diwe KC, Aguocha JK, Enwere OO, et al. Prevalence and Socio-demographic Determinants of Depression among Patients Attending HIV/AIDS Clinic in a Teaching Hospital in Imo State, Nigeria. 3: American Journal of Medical Sciences and Medicine. Science and Education Publishing; 2016. pp. 106–12.

[7] Iloh GUP, Aguocha GU, Amadi AN, Chukwuonye ME (2018). Depression among ambulatory adult patients in a primary care clinic in southeastern Nigeria. Niger Postgrad Med J.

[8] Salihu AS, Udofia O (2016). Prevalence and Associated Factors of Depression among General Outpatients in a Tertiary Institution in Kano, North-Western Nigeria. Open J Psychiatry Sci Res Publishing.

[9] Owolabi LF, Owolabi SD, Taura AA, Alhaji ID, Ogunniyi A (2019). Prevalence and burden of epilepsy in Nigeria: A systematic review and meta-analysis of community-based door-to-door surveys. Epilepsy Behav.

[10] Jatau AI, Sha’aban A, Gulma KA, Shitu Z, Khalid GM, Isa A (2021). The Burden of Drug Abuse in Nigeria: A Scoping Review of Epidemiological Studies and Drug Laws. Public Health Rev.

[11] Okeke AO, Igwe MN, Abamara NC, Nweke CE. Poverty and Mental Health in Nigeria. social Science Research [Internet]. 2018 [cited 2020 Jul 25];4. Available from: https://www.journals.aphriapub.com/index.php/SSR/article/view/596.

[12] World Health Organization (2006). Ministry of Health Nigeria. World Health Organization - Assessment Instrument for Mental Health Systems (WHO-AIMS) Report on Mental Health System in Nigeria.

[13] Soroye MO, Oleribe OO, Taylor-Robinson SD (2021). Community Psychiatry Care: An Urgent Need in Nigeria. J Multidiscip Healthc.

[14] Anyebe EE, Olisah VO, Garba SN, Amedu M (2019). Current Status of Mental Health Services at the Primary Healthcare Level in Northern Nigeria. Adm Policy Ment Health.

[15] Gureje O, Lasebikan VO, Ephraim-Oluwanuga O, Olley BO, Kola L (2005). Community study of knowledge of and attitude to mental illness in Nigeria. Br J Psychiatry Camb Univ Press.

[16] Sheikh TL, Adekeye O, Olisah VO, Mohammed A (2015). Stigmatisation of mental illness among employees of a Northern Nigerian University. Niger Med J.

[17] Abdulmalik J, Olayiwola S, Docrat S, Lund C, Chisholm D, Gureje O (2019). Sustainable financing mechanisms for strengthening mental health systems in Nigeria. Int J Mental Health Syst.

[18] Menberu M, Mekonen T, Azale T, Ayano G, Yimer S, Getnet A (2018). Health care seeking behavior for depression in Northeast Ethiopia: depression is not considered as illness by more than half of the participants. Ann Gen Psychiatry.

[19] Kane JC, Elafros MA, Murray SM, Mitchell EMH, Augustinavicius JL, Causevic S (2019). A scoping review of health-related stigma outcomes for high-burden diseases in low- and middle-income countries. BMC Med.

[20] Ikwuka U, Galbraith N, Manktelow K, Chen-Wilson J, Oyebode F, Muomah RC (2016). Ideological vs. Instrumental Barriers to Accessing Formal Mental Health care in the Developing World: Focus on South-eastern Nigeria. J Health Care Poor Underserved.

[21] Corrigan PW, Watson AC, Barr L (2006). The self-stigma of mental illness: Implications for self-esteem and self-efficacy. Journal of Social and Clinical Psychology.

[22] Corrigan PW, Morris SB, Michaels PJ, Rafacz JD, Rüsch N (2012). Challenging the public stigma of mental illness: a meta-analysis of outcome studies. Psychiatr Serv.

[23] Okpalauwaekwe U, Mela M, Oji C. Knowledge of and Attitude to Mental Illnesses in Nigeria: A Scoping Review. Integrative Journal of Global Health [Internet]. 2017 [cited 2020 Feb 16];1. Available from: http://www.imedpub.com/abstract/knowledge-of-and-attitude-to-mental-illnesses-in-nigeria-a-scoping-review-18642.html.

[24] Obayi NOK, Asogwa F, Ugwunna N (2017). Universal Health Coverage and Healthy Living in South-East Nigeria: How Far with Mental Health?. Open J Psychiatry Sci Res Publishing.

[25] Frank AF, Gunderson JG (1990). The role of the therapeutic alliance in the treatment of schizophrenia. Relationship to course and outcome. Arch Gen Psychiatry.

[26] Totura CMW, Fields SA, Karver MS (2017). The Role of the Therapeutic Relationship in Psychopharmacological Treatment Outcomes: A Meta-analytic Review. PS Am Psychiatric Publishing.

[27] Adewuya AO, Ola BA, Coker O, Atilola O, Fasawe A, Ajomale T (2019). A stepped care intervention for non-specialist health workers’ management of depression in the Mental Health in Primary Care (MeHPriC) project, Lagos, Nigeria: A cluster randomised controlled trial. Gen Hosp Psychiatry.

[28] Coker AO, Olugbile OB, Oluwatayo O. Integration of mental healthcare into primary healthcare in Lagos, Nigeria: the way forward. Healthcare in Low-resource Settings [Internet]. 2015 [cited 2021 Dec 30];3. Available from: https://www.pagepressjournals.org/index.php/hls/article/view/hls.2015.3786.

[29] Adebowale T, Umukoro OL, Gater R, Akinhanmi A, Ogunlesi A, Helme C (2014). Evaluation of a mental health training course for primary health care workers in Ogun State, South West, Nigeria. Afr J Psychiatry (South Africa).

[30] Gureje O, Abdulmalik J, Kola L, Musa E, Yasamy MT, Adebayo K. Integrating mental health into primary care in Nigeria: report of a demonstration project using the mental health gap action programme intervention guide. BMC Health Services Research [Internet]. 2015 [cited 2020 Feb 15];15. Available from: https://link.gale.com/apps/doc/A541448122/AONE?u=29002&sid=zotero&xid=f93840c3. 10.1186/s12913-015-0911-3 PMC4475323 26094025

[31] Federal Ministry of Health Abuja. The National Mental Health Policy for Nigeria. 1991.

[32] Saraceno B, van Ommeren M, Batniji R, Cohen A, Gureje O, Mahoney J (2007). Barriers to improvement of mental health services in low-income and middle-income countries. Lancet.

[33] Nigeria Federal Ministry of Health. National Policy for Mental Health Service Delivery 2013 [Internet]. Abuja N. 2013 Aug. Available from: http://cheld.org/wp-content/uploads/2015/02/national_policy_for_mental_health_service_delivery__2013_.pdf.

[34] Ryan GK, Nwefoh E, Aguocha C, Ode PO, Okpoju SO, Ocheche P (2020). Partnership for the implementation of mental health policy in Nigeria: a case study of the Comprehensive Community Mental Health Programme in Benue State. Int J Ment Health Syst.

[35] World Health Organization. WHO | Sustainable Development Goals (SDGs) [Internet]. WHO. World Health Organization; [cited 2020 Jul 25]. Available from: http://www.who.int/sdg/en/.

[36] Hoeft TJ, Fortney JC, Patel V, Unützer J (2018). Task-Sharing Approaches to Improve Mental Health Care in Rural and Other Low-Resource Settings: A Systematic Review. J Rural Health.

[37] World Health Organization. mhGAP: Mental Health Gap Action Programme: scaling up care for mental, neurological and substance use disorders [Internet]. 2008. Available from: https://apps.who.int/iris/handle/10665/43809. 26290926

[38] World Health Organization. mhGAP Intervention Guide Version 2.0 [Internet]. 2016. Available from: https://www.who.int/publications-detail/mhgap-intervention-guide---version-2.0.

[39] Keynejad R, Spagnolo J, Thornicroft G (2021). WHO mental health gap action programme (mhGAP) intervention guide: updated systematic review on evidence and impact. Evidence-Based Mental Health Royal College of Psychiatrists.

[40] Fetters MD, Curry LA, Creswell JW (2013). Achieving Integration in Mixed Methods Designs—Principles and Practices. Health Serv Res.

[41] NPHCDA. NPHCDA - National Primary Health Care Development Agency [Internet]. NPHCDA. [cited 2020 Aug 18]. Available from: https://nphcda.gov.ng/.

[42] Ogbonna B, Nwako C. Essential Drugs Revolving Fund Scheme in Nigeria; from the Edge of a Precipice towards Sustainability. Journal of Advances in Medical and Pharmaceutical Sciences. 8:1–8.

[43] Wolff G, Pathare S, Craig T, Leff J (1996). Community knowledge of mental illness and reaction to mentally ill people. Br J Psychiatry.

[44] Taylor SM, Dear MJ (1981). Scaling community attitudes toward the mentally ill. Schizophr Bull.

[45] The World Psychiatric Association. The WPA global programme to reduce stigma and discrimination because of schizophrenia [Internet]. 2005. Available from: https://www.open-the-doors.com/english/media/Training_8.15.05.pdf.

[46] Iheanacho T, Marienfeld C, Stefanovics E, Rosenheck RA (2014). Attitudes toward mental illness and changes associated with a brief educational intervention for medical and nursing students in Nigeria. Acad Psychiatry.

[47] Iheanacho T, Kapadia D, Ezeanolue CO, Osuji AA, Ogidi AG, Ike A (2016). Attitudes and beliefs about mental illness among church-based lay health workers: experience from a prevention of mother-to-child HIV transmission trial in Nigeria. Int J Cult Mental Health Routledge.

[48] Iheanacho T, Stefanovics E, Makanjuola V, Marienfeld C, Rosenheck R (2014). Medical and nursing students’ attitudes to people with mental illness in Nigeria: a tale of two teaching hospitals. Int Psychiatry Camb Univ Press.

[49] Damschroder LJ, Aron DC, Keith RE, Kirsh SR, Alexander JA, Lowery JC (2009). Fostering implementation of health services research findings into practice: a consolidated framework for advancing implementation science. Implement Sci.

[50] Clarke V, Braun V. Thematic Analysis. In: Teo T, editor. Encyclopedia of Critical Psychology [Internet]. New York, NY: Springer; 2014 [cited 2021 Dec 31]. p. 1947–52. Available from: 10.1007/978-1-4614-5583-7_311.

[51] The Federal Ministry of Health Abuja, Nigeria. Essential Drugs List - Fourth Revision 2003 - Federal Republic of Nigeria [Internet]. 2003. Available from: https://www.who.int/selection_medicines/country_lists/nga_2003.pdf?ua=1.

[52] Farkas M (2007). The vision of recovery today: what it is and what it means for services. World Psychiatry.

[53] Mutiso VN, Pike K, Musyimi CW, Rebello TJ, Tele A, Gitonga I, et al. Feasibility of WHO mhGAP-intervention guide in reducing experienced discrimination in people with mental disorders: a pilot study in a rural Kenyan setting. Epidemiology and Psychiatric Sciences. Cambridge University Press; 2019;28:pp. 156–67. 10.1017/S2045796018000264 PMC6999029 29862937

[54] Kohrt BA, Turner EL, Rai S, Bhardwaj A, Sikkema KJ, Adelekun A (2020). Reducing mental illness stigma in healthcare settings: Proof of concept for a social contact intervention to address what matters most for primary care providers. Soc Sci Med.

[55] Li J, Fan Y, Zhong H-Q, Duan X-L, Chen W, Evans-Lacko S (2019). Effectiveness of an anti-stigma training on improving attitudes and decreasing discrimination towards people with mental disorders among care assistant workers in Guangzhou, China. Int J Ment Health Syst.

[56] Eaton J, Agomoh AO (2008). Developing mental health services in Nigeria: the impact of a community-based mental health awareness programme. Soc Psychiatry Psychiatr Epidemiol.

[57] Faregh N, Lencucha R, Ventevogel P, Dubale BW, Kirmayer LJ (2019). Considering culture, context and community in mhGAP implementation and training: challenges and recommendations from the field. Int J Ment Health Syst.

[58] Pratt B, de Vries J (2018). Community engagement in global health research that advances health equity. Bioethics.

[59] Gureje O, Oladeji BD, Montgomery AA, Bello T, Kola L, Ojagbemi A (2019). Effect of a stepped-care intervention delivered by lay health workers on major depressive disorder among primary care patients in Nigeria (STEPCARE): a cluster-randomised controlled trial. The Lancet Global Health.

[60] Twisselmann B (2005). Africa’s medical brain drain: Summary of responses to recent editorial on stopping Africa’s brain drain. BMJ Br Med J Publishing Group.

[61] Onwujiariri CM, Nwachi CC, Nkwocha EE (2017). Rural Healthcare Service Provision and Implication in Rural Development of Imo State. Nigeria IJLERA.

[62] U-Report. U-Report Nigeria - Imo State [Internet]. U-Report. 2018 [cited 2020 Mar 12]. Available from: https://nigeria.ureport.in/opinion/2970/.

[63] National Population Commission. Nigeria Demographic and Health Survey 2018 [Internet]. Abuja N. 2019 Oct. Available from: https://www.dhsprogram.com/pubs/pdf/FR359/FR359.pdf.

[64] WHO Africa. Imo State Health Insurance Scheme on course to achieving universal health coverage [Internet]. WHO - Regional Office for Africa. 2019 [cited 2020 Mar 12]. Available from: https://www.afro.who.int/news/imo-state-health-insurance-scheme-course-achieving-universal-health-coverage.

[65] Onwujekwe O, Hanson K, Uzochukwu B (2012). Examining Inequities in Incidence of Catastrophic Health Expenditures on Different Healthcare Services and Health Facilities in Nigeria. PLOS ONE Public Library of Science.

[66] Agboola AA, Esan OT, Afolabi OT, Soyinka TA, Oluwaranti AO, Adetayo A (2018). Economic burden of the therapeutic management of mental illnesses and its effect on household purchasing power. PLOS ONE Public Library of Science.

[67] Oloniniyi IO, Akinsulore A, Aloba OO, Mapayi BM, Oginni OA, Makanjuola R (2019). Economic Cost of Schizophrenia in a Nigerian Teaching Hospital. J Neurosci Rural Pract.

