# Supplementary material for: Integrating mental health into primary care: evaluation of the Health Action for Psychiatric Problems In Nigeria including Epilepsy and SubstanceS (HAPPINESS) pilot project
Source: BMC Health Serv Res. 2022 Mar 12;22:333. doi: 10.1186/s12913-022-07703-1 (PMC8917687; doi:10.1186/s12913-022-07703-1)
Supplement: Supplementary file 1 — Additional file 1. The HAPPINESS Project Standard Operating Procedure (SOP). SOP for clinical record-keeping and reporting for primary care nurses and community health workers. [file 12913_2022_7703_MOESM1_ESM.pdf]

The HAPPINESS Project
**STANDARD OPERATING PROCEDURE**
**CLINICAL RECORD-KEEPING AND REPORTING FOR PRIMARY CARE NURSES AND COMMUNITY HEALTH WORKERS**

Effective record-keeping and reporting are crucial for accountability, transparency and good clinical decision-making. It also demonstrates the value of services for the purposes of research, evaluation and advocacy. This SOP will help Community Psychiatric Nurses (CPNs) involved in the HAPPINESS Project to set up an office record-keeping and reporting system that can accommodate the many unique needs of mental health service users, providers, donors, and other stakeholders.

These standard operating procedures (SOPs) are designed as a reference to help you get started. They cannot substitute for adequate training and supervision. They are designed to help you to better understand what is expected of you in your administrative role as a CPN. It contains a directory of the records and tools, equipment you will need to access in order to use and to store them.

SECTION 1. What you need

- Appointment cards (Appendix 1):
- Daily register (Appendix 2)
  - **New client register**
  - **Existing client register**
- Monthly reporting forms (Appendix 3):
- Patient files with logs (Appendix 4)
- Enrolment forms(Appendix 5)
- Follow-up forms (Appendix 6)
- 6-month review forms (Appendix 7)
- Referral register (Appendix 8)
- Referral forms (Appendix 9)
- GAF scoring tool (Appendix 10)
- Monthly register tally sheet (Appendix 11)

SECTION 2. Equipment checklist:

- Secure office space:

- Stapler and scissors:
- Pens.
- Additional files/envelopes:

SECTION 3. Getting organized

A. ***Storing clinical records***

- Keep all the necessary clinical forms for one patient in one file
- Do not throw away clinical records, even if the patient has been lost to follow-up or referred to another clinic to continue services
- If you end up with two files on the same patient (e.g., if you accumulate too many records to fit in a single file), clearly label on the front of the first file "file 1 of 2", and on the second file "file 2 of 2". Be certain to include the patient ID on each of the two files

B. Recording your services

Enrolling a new client

1. When a new client arrives at your services, you should first **open a *new patient file*** by filling out a patient file log (Appendix 4) with the following:

a. Patient ID no.

b. Clinic name

c. Visit date (today's date, recorded in row 1, the shaded row with "Enrollment" as the "Visit Type")

2. If this ***file log*** is not already printed on the cover of the patient file, staple it to the inside cover of the file, and write the patient ID no. clearly on the outside of the file.

3. ***Complete an enrollment form,*** being certain to answer every question and label each page with the patient ID no., as instructed in the guide to patient forms. You might also want to refer to the GAF scoring tool to help you complete this section of the tool. When complete, place the enrollment form inside the patient file.

4. ***Issue an appointment card*** to the new client, by completing the following:

a. Clinic name*

b. Patient name*

c. Patient ID no.*

d. Any crucial notes (e.g. clinic phone number in case of emergency)

e. Today's date* (next to "1" on the reverse side of the appointment card)

f. Next appointment date (next to "2" on the reverse side of the appointment card)

g. Copy the required information from the enrollment form to the ***new client register***, in your daily register.

C. ***Following up on an existing client***

1. For any existing client who arrives at your services, you should take the client's appointment card and use the patient ID no. recorded on the card to ***locate the patient's file***.*

2. ***Examine the patient file log***. If it has been less than six months since the client's last 6-month review or enrollment visit, update the log with the following:

a. Visit date (today's date)

b. Visit type (tick "follow-up")

3. ***Complete a follow-up form***, being certain to answer every question and label each page with the patient ID no. ***Be sure to note any changes to the prescription, including dosage and cost***, since the last visit. When complete, place the follow-up form inside the patient file.

4. ***Update the appointment card for the client***, by adding the next scheduled appointment date in the next available row on the reverse side of the card.

5. ***Add the required information to the existing client register of the daily register***, using information from the patient's enrollment form and the follow-up form you just completed. For "Visit Type," look back to the patient file log to see whether this is the patient's first follow-up visit this month. If it is, tick the cell for "First follow-up of the month". If it isn't, tick the other cell for "Extra follow-up this month".

D. 6-month review of on an existing client

1. For any existing client who arrives at your services, you should take the client's appointment card and use the patient ID no. recorded on the card to ***locate the patient's file.***

2. Examine the patient file log. If it has been six months or more since the client's last 6-month review or enrollment visit, ***update the log*** with the following:

c. Visit date (today's date)

d. Visit type (tick "6-Month Review")

3. ***Complete a 6-month review form***, being certain to answer every question and label each page with the patient ID no. You might also want to refer to the ***GAF scoring tool*** (Appendix 10) to help you complete this section of the tool. When complete, place the 6-month review form inside the patient file.

4. ***Update the appointment card for the client***, by adding the next scheduled appointment date in the next available row on the reverse side of the card.

5. ***Add the required information to the daily register using information from the patient's enrollment form and the 6-month review form you just completed***. For "Visit Type," look back to the patient file log to see whether this is the patient's first visit this month. If it is, tick the cell for "First follow-up of the month". If it isn't, tick the other cell for "Extra follow-up this month".

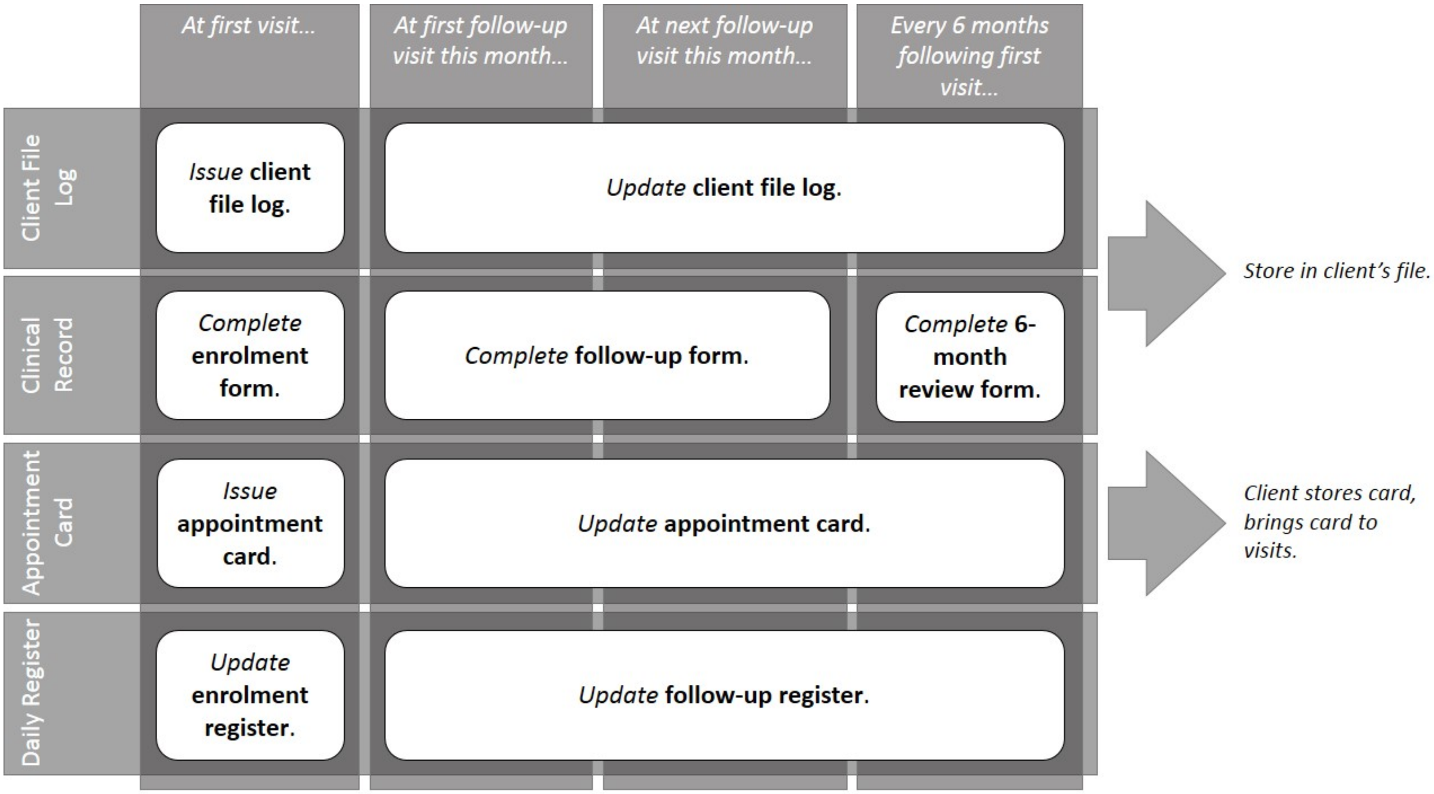

Figure 1. How clinical record keeping responsibilities change between first visit and follow-up visits

E. Filling prescriptions

1. Refer to the most recent form completed for the client in the client's patient file, in order to see the prescription, dosage, and total cost.

2. Fill the prescription with the following information:

a. Patient ID no.

b. Payer’s name

c. Today’s date

F. Referring clients

1. Whether this is an enrollment, follow-up, or 6-month review visit, you may wish to refer a patient to additional services. Each of the relevant forms includes a section in which you should record the referral.

2. The referral must also be ***recorded in the “Referrals” section of the daily register*** as an “outgoing referral”.

3. Complete the top-half of a referral form, and give the full form (top and bottom halves intact) to the patient to take to the receiving facility. ***Instruct the patient*** to give the whole referral form to the receiving facility, and to bring the bottom-half only back to your clinic. The top-half remains with the receiving facility.

4. ***Make a new entry on the “Register of Referrals OUT” table of the referral register***, recording this referral. When the client does eventually return with the bottom-half (the “back-referral” section), write the date of receipt in the “Date back referral received” column of the register.

5. ***File the returned “back-referral” section of the referral form*** in the patient file, using the patient ID no. to locate the appropriate file.

G. Receiving referrals

1. When a new client comes to the clinic with a referral form, first proceed with the general process for ***“Enrolling a new client”*** (Section 3.B).

2. Ensure that the referral was ***recorded in the “Referrals” section of the daily register*** as an “incoming referral”.

3. Make a ***new entry on the “Register of Referrals IN” table*** of the referral register, recording this referral.

4. After the client has been enrolled, ***complete the bottom-half (“back referral” section)*** of the referral form, and ***detach it from the top section***. ***Return the bottom half to the patient*** to bring back to the initiating facility. Store the top-half in the new patient file.

5. ***If all of the record-keeping work cannot be done each day?*** Do not “cut corners” with record-keeping in order to save time. If you find you must work late hours in order to serve all your

clients and update their records daily, ***speak with your supervisor about getting assistance***. The items marked with an asterisk* can be "task-shifted" to a less-specialized health worker, as long as he or she is well-trained and supervised. Your supervisor may also observe a day in the clinic with you and make suggestions about strategies to save time and improve efficiency.

6. *What to do if an existing client shows up without a card.*

Do not assign him or her a new patient ID no. or create a new file. You can still ***locate the patient ID no. to retrieve the existing file***. Ask the client approximately when he or she last attended the clinic, and scan your daily register from around that time period to search for the client's name.* Each entry in the register should have the date, client's name, and patient ID no. listed, so you should then be able to retrieve the correct patient ID no. and corresponding file. **Issue a new appointment card,** if necessary, and ***urge the client to bring it to the next appointment.***

7. Who fills out the Data Entry Date on the file log?

The clinical officer or another supervisor responsible for data entry will fill this section after entering patient data electronically from the files in the form.

8. What do you do if not sure about how to record something?

Never skip a question on any form unless the form specifically advises you to (e.g. on a question that says, "Only complete for epilepsy patients" when you are recording information for a patient without epilepsy). These SOPs and the supporting materials provided should be able to help guide you, but if you're still uncertain, or if you feel that the options provided don't adequately reflect the information you wish to record (e.g. if you've prescribed a drug that isn't listed on the list of drug options available to tick), please consult your supervisor as soon as possible. You may have identified an issue with the tools provided that we need to address in order to ensure that they are as clear and as useful as possible to CPNs.

SECTION 4. Reporting your services

A. Routine Reporting: HIS data

1. ***Each month, tally all of the information recorded in the previous month in the new client register of the daily register,*** by counting the total number of ticks in each column. Use the monthly register tally sheet to help you. Then use this information to ***fill in the appropriate sections in the monthly reporting form, including***:

a. Number of new clients served, disaggregated by age and sex

b. Number of new clients served, disaggregated by CGI-S score

c. Number of new clients served, disaggregated by diagnosis

2. Each month, ***tally all of the information recorded from each unique follow-up client in the existing client register,*** by using the monthly register tally sheet to separate the information collected from clients' first follow-up visit of the month from information that may have been collected on subsequent visits. Then use the information tallied up from first visits only to ***fill in the appropriate sections in the monthly reporting form,*** including:

a. Number of follow-up clients served, disaggregated by age and sex

b. Number of follow-up clients served, disaggregated by CGI-S score

c. Number of follow-up clients served, disaggregated by diagnosis

3. Each month, ***tally up the total number of incoming and outgoing referrals from both the new client register and the existing client register.*** Add these together to fill in the last two sections of the monthly reporting form:

a. Number of clients referred into the facility

b. Number of clients referred out of the facility

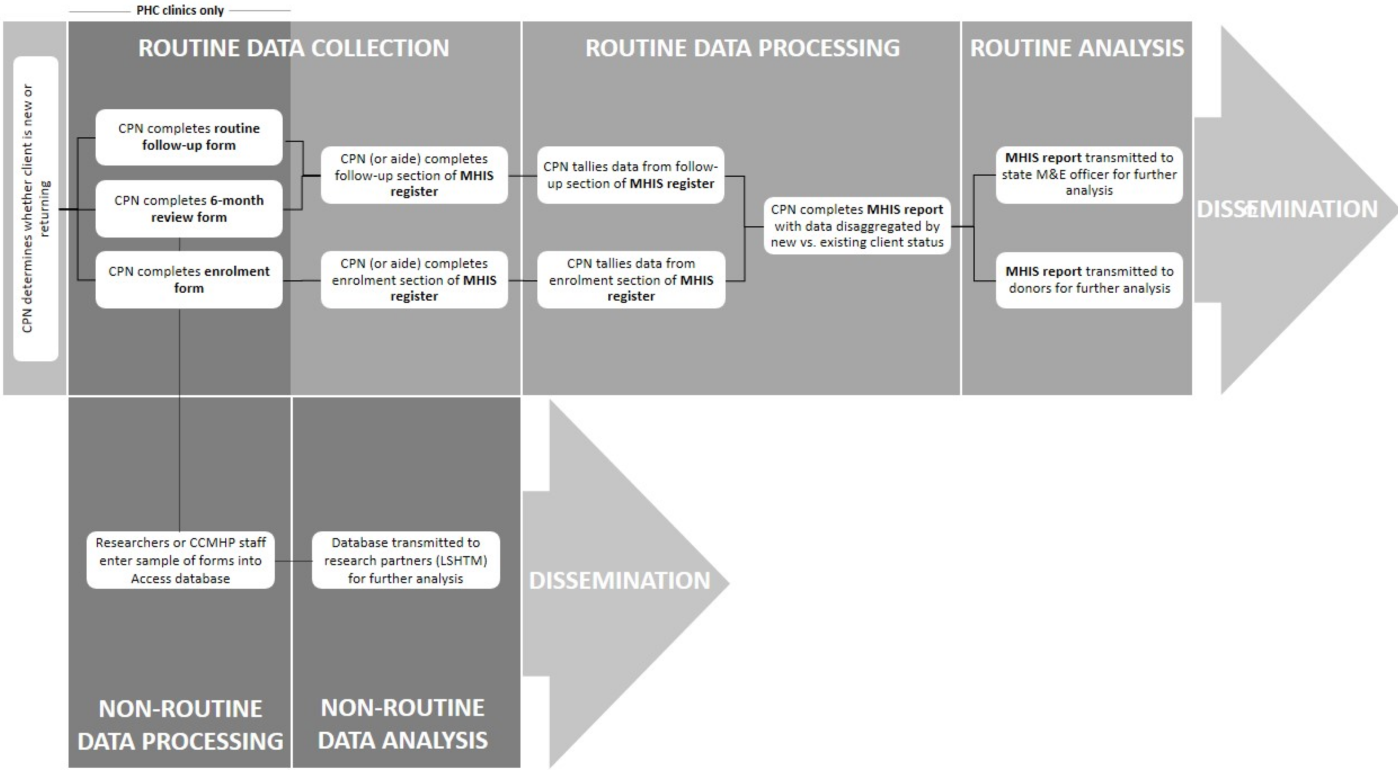

Figure 2. How HIS reporting fits within the M&E strategy

4. ***Why there is need to report HIS data each month***

In order to ensure timely reporting of key indicators through State channels, HIS data is aggregated on a monthly basis by the CPN and submitted to both the health center management and to the project. Aggregating the data not only ensures transparency, it also enables you to be responsive to important trends and changes to improve the quality of your services.

For example, a CPN who finds she has received few referrals may decide she needs to visit other health centers in the area to make them aware of her services. Another CPN who notices he receives mostly clients with psychosis will want to request a larger supply of anti-psychotics, and further conduct community outreach to increase detection of clients with other diagnoses like depression or epilepsy.

5. What happens to all the other client data I have recorded in my forms?

The forms you include in each patient file include a lot of rich data that can help you to remember key information about each client, make clinical decisions appropriate to his or her needs, and judge his or her progress. This information also enables your supervisor to help advise you on a case-by-case basis.

Eventually, this information will also be aggregated and analyzed in order to evaluate the program as a whole. As you can see in Figure 2, shown above, monthly HIS reporting is the routine process we rely on to monitor services routinely. Less routinely, partners will use the rich data you've collected to evaluate the impact this service has on patient outcomes and help build the evidence base for community mental health in Cameroon.

6. What to do if you think a mistake has been made?

Be open with your supervisor about any errors you think you may have made in recording or reporting data. The feedback you provide will help to inform further refinement.

7. What will my supervisor want to see?

One of the roles of your supervisor is to support you both in the actual provision of services and also in how you record and report your services. During supervision visits, he or she will want to see the daily register, monthly report, and a selection of patient files—especially any about which you have questions you would like to ask.

APPENDIX 1

APPOINTMENT CARD

Please present this card to the clinic staff.

**Clinic Name:**							
**Patient Name:**							
**Patient No:**							
**Notes:**							

Appointments

	Date		Date
1		19	
2		20	
3		21	
4		22	
5		23	
6		24	
7		25	
8		26	
9		27	
10		28	
11		29	
12		30	
13		31	
14		32	
15		33	
16		34	
17		35	
18		36	

APPENDIX 2
PATIENT NUMBER

COMMUNITY MENTAL HEALTH CLINIC
PATIENT FILE
Clinic: ____

#	Visit Date	Visit Type<br><br>(Tick one)	Data Entry Date<br><br>(For clinical officer use only)
1		-Enrollment-	
2		☐ Follow-Up ☐ 6-Month Review	
3		☐ Follow-Up ☐ 6-Month Review	
4		☐ Follow-Up ☐ 6-Month Review	
5		☐ Follow-Up ☐ 6-Month Review	
6		☐ Follow-Up ☐ 6-Month Review	
7		☐ Follow-Up ☐ 6-Month Review	
8		☐ Follow-Up ☐ 6-Month Review	
9		☐ Follow-Up ☐ 6-Month Review	
10		☐ Follow-Up ☐ 6-Month Review	
11		☐ Follow-Up ☐ 6-Month Review	
12		☐ Follow-Up ☐ 6-Month Review	
13		☐ Follow-Up ☐ 6-Month Review	
14		☐ Follow-Up ☐ 6-Month Review	
15		☐ Follow-Up ☐ 6-Month Review	
16		☐ Follow-Up ☐ 6-Month Review	
17		☐ Follow-Up ☐ 6-Month Review	
18		☐ Follow-Up ☐ 6-Month Review	
19		☐ Follow-Up ☐ 6-Month Review	

20		☐ Follow-Up ☐ 6-Month Review	
21		☐ Follow-Up ☐ 6-Month Review	
22		☐ Follow-Up ☐ 6-Month Review	
23		☐ Follow-Up ☐ 6-Month Review	
24		☐ Follow-Up ☐ 6-Month Review	
25		☐ Follow-Up ☐ 6-Month Review	
26		☐ Follow-Up ☐ 6-Month Review	
27		☐ Follow-Up ☐ 6-Month Review	
28		☐ Follow-Up ☐ 6-Month Review	
29		☐ Follow-Up ☐ 6-Month Review	
30		☐ Follow-Up ☐ 6-Month Review	
